# Early and late outcomes after minimally invasive direct coronary artery bypass vs. full sternotomy off-pump coronary artery bypass grafting

**DOI:** 10.3389/fcvm.2024.1298466

**Published:** 2024-02-21

**Authors:** Mohammad Sharaf, Armin Zittermann, Jakub Sunavsky, Tomasz Gilis-Januszewski, Sebastian V. Rojas, Julia Götte, Dragan Opacic, Darko Radakovic, Georges El-Hachem, Artyom Razumov, Andre Renner, Jan F. Gummert, Marcus-André Deutsch

**Affiliations:** Department of Thoracic and Cardiovascular Surgery, Heart- and Diabetes Center NRW, Ruhr-University Bochum, Bad Oeynhausen, Germany

**Keywords:** coronary artery bypass grafting, myocardial revascularization, off-pump coronary artery bypass grafting, MIDCAB, full sternotomy

## Abstract

**Objectives:**

Minimally-invasive direct coronary artery bypass (MIDCAB) is a less-invasive alternative to full sternotomy off-pump coronary artery bypass (FS-OPCAB) revascularization of the left anterior descending artery (LAD). Some studies suggested that MIDCAB is associated with a greater risk of graft occlusion and repeat revascularization than FS-OPCAB LIMA-to-LAD grafting. Data comparing MIDCAB to FS-OPCAB with regard to long-term follow-up is scarce. We compared short- and long-term results of MIDCAB vs. FS-OPCAB revascularization over a maximum follow-up period of 10 years.

**Patients and methods:**

From December 2009 to June 2020, 388 elective patients were included in our retrospective study. 229 underwent MIDCAB, and 159 underwent FS-OPCAB LIMA-to-LAD grafting. Inverse probability of treatment weighting (IPTW) was used to adjust for selection bias and to estimate treatment effects on short- and long-term outcomes. IPTW-adjusted Kaplan–Meier estimates by study group were calculated for all-cause mortality, stroke, the risk of repeat revascularization and myocardial infarction up to a maximum follow-up of 10 years.

**Results:**

MIDCAB patients had less rethoracotomies (*n* = 13/3.6% vs. *n* = 30/8.0%, *p* = 0.012), fewer transfusions (0.93 units ± 1.83 vs. 1.61 units ± 2.52, *p* < 0.001), shorter mechanical ventilation time (7.6 ± 4.7 h vs. 12.1 ± 26.4 h, *p* = 0.005), and needed less hemofiltration (*n* = 0/0% vs. *n* = 8/2.4%, *p* = 0.004). Thirty-day mortality did not differ significantly between the two groups (*n* = 0/0% vs. *n* = 3/0.8%, *p* = 0.25). Long-term outcomes did not differ significantly between study groups. In the FS-OPCAB group, the probability of survival at 1, 5, and 10 years was 98.4%, 87.8%, and 71.7%, respectively. In the MIDCAB group, the corresponding values were 98.4%, 87.7%, and 68.7%, respectively (RR1.24, CI0.87–1.86, *p* = 0.7). In the FS group, the freedom from stroke at 1, 5, and 10 years was 97.0%, 93.0%, and 93.0%, respectively. In the MIDCAB group, the corresponding values were 98.5%, 96.9%, and 94.3%, respectively (RR0.52, CI0.25–1.09, *p* = 0.06). Freedom from repeat revascularization at 1, 5, and 10 years in the FS-OPCAB group was 92.2%, 84.7%, and 79.5%, respectively. In the MIDCAB group, the corresponding values were 94.8%, 90.2%, and 81.7%, respectively (RR0.73, CI0.47–1.16, *p* = 0.22).

**Conclusion:**

MIDCAB is a safe and efficacious technique and offers comparable long-term results regarding mortality, stroke, repeat revascularization, and freedom from myocardial infarction when compared to FS-OPCAB.

## 1 Introduction

Minimally invasive direct coronary artery bypass (MIDCAB) grafting is a sternal-sparing procedure to achieve surgical revascularization of the anterior wall of the left ventricle using the left internal mammary artery (LIMA) through a lateral thoracotomy. Common complications of the median sternotomy and the use of cardiopulmonary bypass (CPB) are avoided. MIDCAB surgery aims to provide patients with reduced postoperative pain, shorter hospital stays, quicker recovery times, and excellent cosmesis (1, 2). Like Off-Pump Coronary Artery Bypass (OPCAB), MIDCAB decreases the risk for CPB-associated systemic inflammatory response, reduces bleeding and need for transfusions, in addition to the reduction to postoperative renal dysfunction and neurological complications when compared to conventional coronary artery bypass grafting (CABG) (3). MIDCAB is most commonly performed in isolated left anterior descending (LAD) disease when percutaneous coronary intervention (PCI) is not advisable, not successful, too complex, or in symptomatic patients who have previously undergone PCI of the LAD and signs in-stent stenosis. Recent meta-analyses have shown MIDCAB superiority over PCI for treating proximal LAD lesions with drug-eluting stents in terms of freedom from repeat revascularization (4–6). Patients with multivessel disease who have already undergone stenting of non-LAD vessels can subsequently have MIDCAB grafting of the LAD (2). Likewise, in patients with multivessel-disease, MIDCAB is increasingly being used in the setting of hybrid revascularization with LIMA-to-LAD grafting and drug-eluting stent (DES) to non-LAD lesions (7–9). One of its most significant advantages is the long-term, event-free survival due to the longevity of the left internal mammary artery graft which remains the standard of care for surgical revascularization of the LAD (2).

However, MIDCAB is a technically demanding procedure and there are ongoing concerns about the ability to accurately perform LIMA harvesting and to complete the coronary anastomosis on a beating heart with both limited surgical access and exposure. Controversially, some studies suggested that, although MIDCAB and Off-Pump Coronary Artery Bypass may be similar in terms of early and mid-term mortality, the minimally invasive approach may have increased long term risks such as graft occlusion and repeat revascularization than OPCAB via sternotomy (10–12). Additionally, there is limited data directly comparing MIDCAB and OPCAB grafting, especially pertaining to the long-term outcomes.

Given the uncertainties regarding risks, benefits, and the limited published long-term outcomes, we aimed to compare the short- and long-term results of MIDCAB vs. Full Sternotomy (FS-OPCAB) revascularization over a maximum follow-up period of 10 years.

## 2 Patients and methods

The study was conducted in accordance with the principles of the Declaration of Helsinki and was approved by the ethics committee of the Ruhr-University-Bochum, Germany. Because of the retrospective study design requirement for written informed consent was waived.

**Figure 1:**
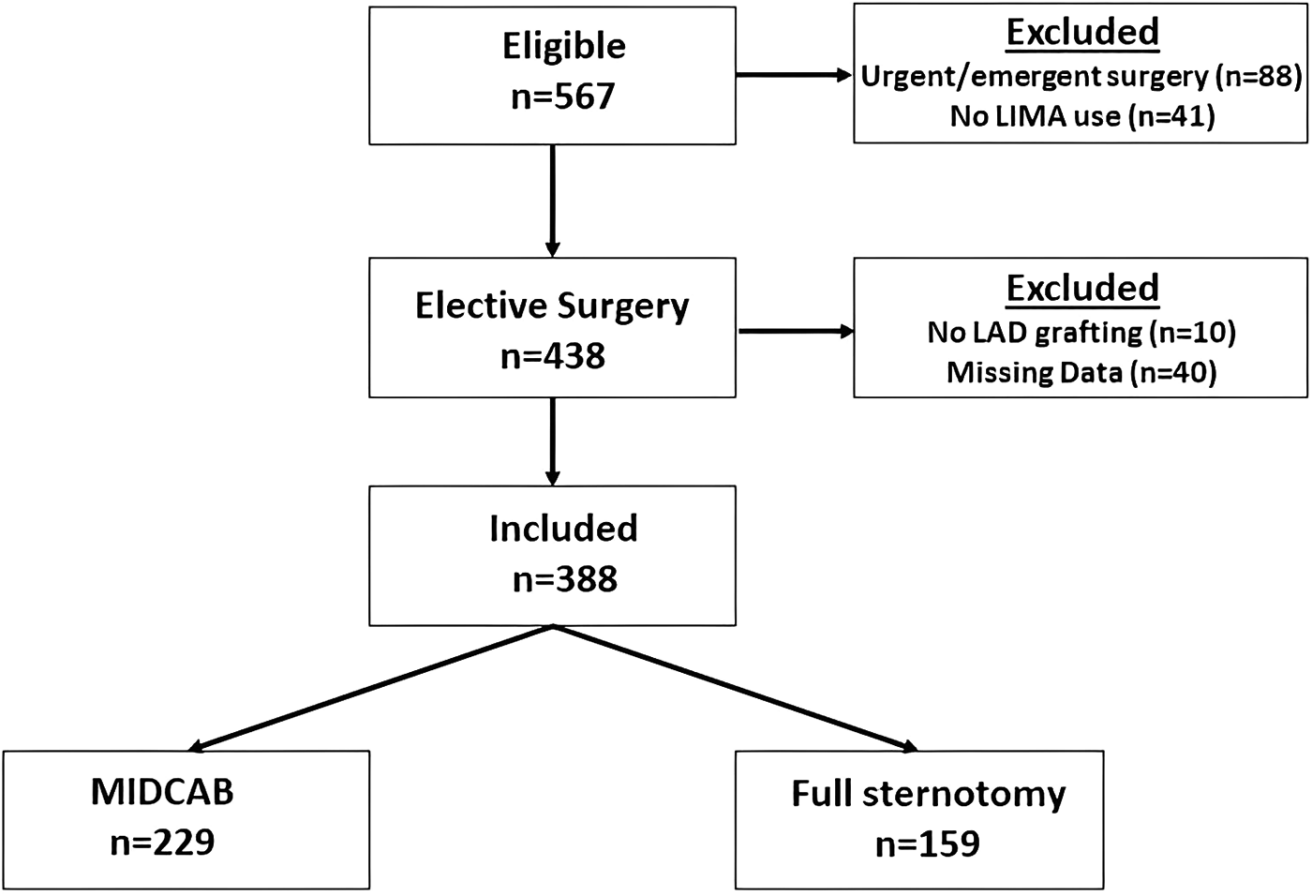
Flow of patients into the final cohort (arrows) or to study exclusion, based on inclusion and exclusion criteria.

From December 2009 to June 2020, all consecutive patients undergoing elective MIDCAB or FS-OPCAB surgery were included. All patients received a LIMA-to-LAD graft either through a small left anterior thoracotomy or via median sternotomy. Patients who underwent sequential grafting of a diagonal branch were also included. Patients requiring additional cardiac surgery or with a history of any previous cardiac surgery were excluded. Hemodynamically instable patients (preoperative cardiogenic shock, or after cardiopulmonary resuscitation) or patients with an emergency status as well as conventional or on pump beating heart procedures were also excluded. As depicted in the study flow chart (Figure 1), a total of 388 patients were included in our retrospective study. Of these patients, 229 underwent LIMA-to-LAD grafting via an antero-lateral minithoracotomy (MIDCAB group), and 159 underwent off-pump myocardial revascularization through a median sternotomy (FS-OPCAB group) within the same time frame. Preoperative, intraoperative, and postoperative data were prospectively recorded in a dedicated database on a routine basis. Clinical data were obtained from a database using the cardiac surgery acquisition program THGQIMS (Münster, Germany). Biochemical parameters were obtained from Lauris (SWISSLAB, Berlin, Germany). The study was performed in accordance with the STrengthening the Reporting of Observational studies in Epidemiology (STROBE) statement (www.strobe-statement.org).

## 3 Surgical techniques and intraoperative anesthetic management

Surgical access choice was left at the surgeon's discretion. MIDCAB patients were treated in a similar manner compared to patients who underwent conventional CABG. Both procedures were performed under general anaesthesia. OPCAB patients were intubated with a regular single-lumen tracheal tube, and double-lumen endotracheal tubes to allow single-lung ventilation of the right lung in were used in MIDCAB patients. Defibrillation electrodes were preoperatively placed by the anaesthesiologist on the back away from the planned incision site. Patients were placed in the supine position, and the left side of the thorax was slightly elevated using a surgical balloon. After the incision, one-lung ventilation on the right side was performed during the entire operation. An 8–9 cm incision was done in the 4th or 5th intercostal space and the pleura was opened. The suspension-type internal mammary artery retraction system (Thoralift, US Surgical Corp.) was placed, and under direct vision, the left internal mammary artery (LIMA) was prepared. Intravenous heparin was given with a dose of 250 IU/kg body weight prior to the division of the LIMA to acheive an activated coagulation time greater than 350 s. The pericardium is then opened over the LAD region and the heart is lightly luxated and elevated using a Vicryl suture to expose the intended area. The heart is then stabilized using a tissue stabilizer (Octopus Tissue Stabilization System, Medtronic, USA). The LIMA-to-LAD is then anastomosed using a 7-0 or 8-0 running polypropylene suture with or without the use of a coronary shunt. The anastomosis is then checked for any bleeding. FS-OPCAB operations were carried out through a median sternotomy. Ultracision or electrocautery were used to harvest the LIMAA deep pericardial traction suture was placed to help with the luxation of the heart. Stabilization of the anterior wall was reached with the Octopus stabilizer (Medtronic Inc, Minneapolis, MN). The LIMA-to-LAD anastomosis was carried out in a standard fashion as described above. Mean flow (MF), pulsatility index (PI), diastolic filling percentage (DF) were recorded using Medistim (Medistim ASA, Oslo, Norway). Heparin was antagonized with Intravenous protamine after completion of the anastomosis. The pericardium is then partially closed. Thoracic drains are placed and the wound is closed.

## 4 Postoperative management

The patients were transferred under general anesthesia to our surgical intensive care unit (ICU). During the admission, all patients received ECG, Chest Xray and blood labs which include arterial blood gases, troponin, and creatinine kinase. The blood labs were repeated every 4 h. When the patients showed hemodynamic cardiac and respiratory stability, they were extubated. After 6 h, the patients received 500 mg intravenous aspirin and were started on continuous intravenous heparin. The patients were transferred to the normal ward after approximately 24 h of intensive care monitoring. The surgical drains were removed on the second postoperative day and patients were then mobilized. An ECG and transthoracic echocardiography were performed on the fifth postoperative day and patients were typically discharged 7–10 days after the procedure.

## 5 Clinical endpoints and follow-up

Mortality, as the primary endpoint, was assessed by using the following sources of information: a regular review of medical records, repeated contacts with the patients or their relatives, contact with family physicians. Secondary endpoints included freedom from myocardial infarction, stroke, or repeat revascularization during follow-up. Myocardial infarction was defined as occurrence of new persistent ST segment changes in addition to an elevation in cardiac troponin values (perioperative: hs-troponin I >10,000 ng/ml, troponin I >10 mg/L; after discharge: hs-troponin I >500 ng/ml, troponin I >0.5 mg/L) with or without evidence of new regional wall motion abnormalities. Coronary angiography was performed in all patients with suspected myocardial infarction. A stroke event was considered present when clinically significant motor, sensory, or cognitive neurological deficit was recorded due to a cerebrovascular event and subsequently was confirmed by imaging modality. Transient ischemic events were not included. Repeat revascularization was defined as post-operative PCI or redo CABG during follow-up. Secondary clinical endpoints were assessed by the same sources used to identify the primary endpoint (exception: registration office). The completeness of follow-up was 99.6%. Mean follow-up length of 5.76 ± 3.13 years.

Because of non-randomized treatment group assignment and to adjust for selection bias, we generated a propensity score (PS) for each patient. For PS generation, we used the multivariable logistic regression model with type of surgery (MIDCAB or full sternotomy) as binary dependent variable. The model comprised the following baseline covariates: age, sex, previous percutaneous coronary intervention, NYHA functional class, stroke, eGFR, carotid artery stenosis, diabetes, smoking, COPD, arterial hypertension, atrial fibrillation, LVEF, 3-vessel disease, left main stenosis, CHA_2_DS_2_ VASC score, and use of statins and aspirin. Mentioned variables were included regardless of their statistical significance. The precision of discrimination and calibration of the PS were analyzed with the c-statistic and the Hosmer–Lemeshow test for goodness of fit. After PS calculation, we applied inverse probability of treatment weighting (IPTW) to reduce the bias of unweighted estimators and adjust for covariates imbalance between the two study groups. The following formula was applied: T/PS + (1—T)/(1—PS), whereby T indicates patient status being 0 in patients with full sternotomy and 1 in patients with MIDCAB surgery. Since the treatment effects obtained using IPTW may be interpreted as causal, this approach has some assumptions, such as exchangeability, no misspecification of the propensity score model, positivity and consistency, that have to be fulfilled (13). To achieve exchangeability, weights were truncated at PS values below 0.10 and above 0.90 (Supplementary Figure S1). To prevent misspecification, 21 important risk factors of cardiac surgery were included in the model and post-weighting balance in covariates was evaluated by using standardized mean differences (SMD). The balance is considered to be satisfactory when the SMD is less than 10%. The assumption of positivity was realized, as there were both exposed and unexposed individuals for every potential confounder. To test consistency, we compared the differences in 1-year and 5-year mortality between the study groups for the years 2009 to 2014 and 2015 to 2020.

The Mann–Whitney *U*-test was applied to compare IPTW-adjusted continuous outcome data such as troponin_max_ or hs troponin_max_, creatinine_max_, use of red blood cell units, duration of mechanical ventilation, intensive care unit stay, and in-hospital stay. Fisher's exact test was used to compare IPTW-adjusted perioperative clinical outcomes such as the need for rethoracotomy, hemofiltration, stent implantation, and wound infection until discharge, 30-day mortality, and the need for readmission for wound infection. Moreover, we generated IPTW-adjusted Kaplan–Meier estimates by study group for the risk of repeat revascularization, stroke, myocardial infarction and overall mortality up to a maximum follow-up of 10 years. Results were compared by the log rank test.

For baseline characteristics, continuous variables are presented as mean with standard deviation. Categorical variables are summarized as percentages and number of observations. Continuous outcome variables are presented as median with 25th and 75th percentiles. Clinical outcome data are presented as relative risks (RRs) and 95% confidence intervals (CIs). *P* values < 0.05 were considered statistically significant. To account for multiple testing, the Benjamini and Hochberg false discovery rate method was considered to adjust the *P* values as previously described (14). The false recovery rate was set at 5%. We performed all analyses using IBM SPSS Statistics version 24 (IBM Corporation, Armonk, NY, USA).

## 6 Results

### 6.1 Baseline characteristics

**Figure 2:**
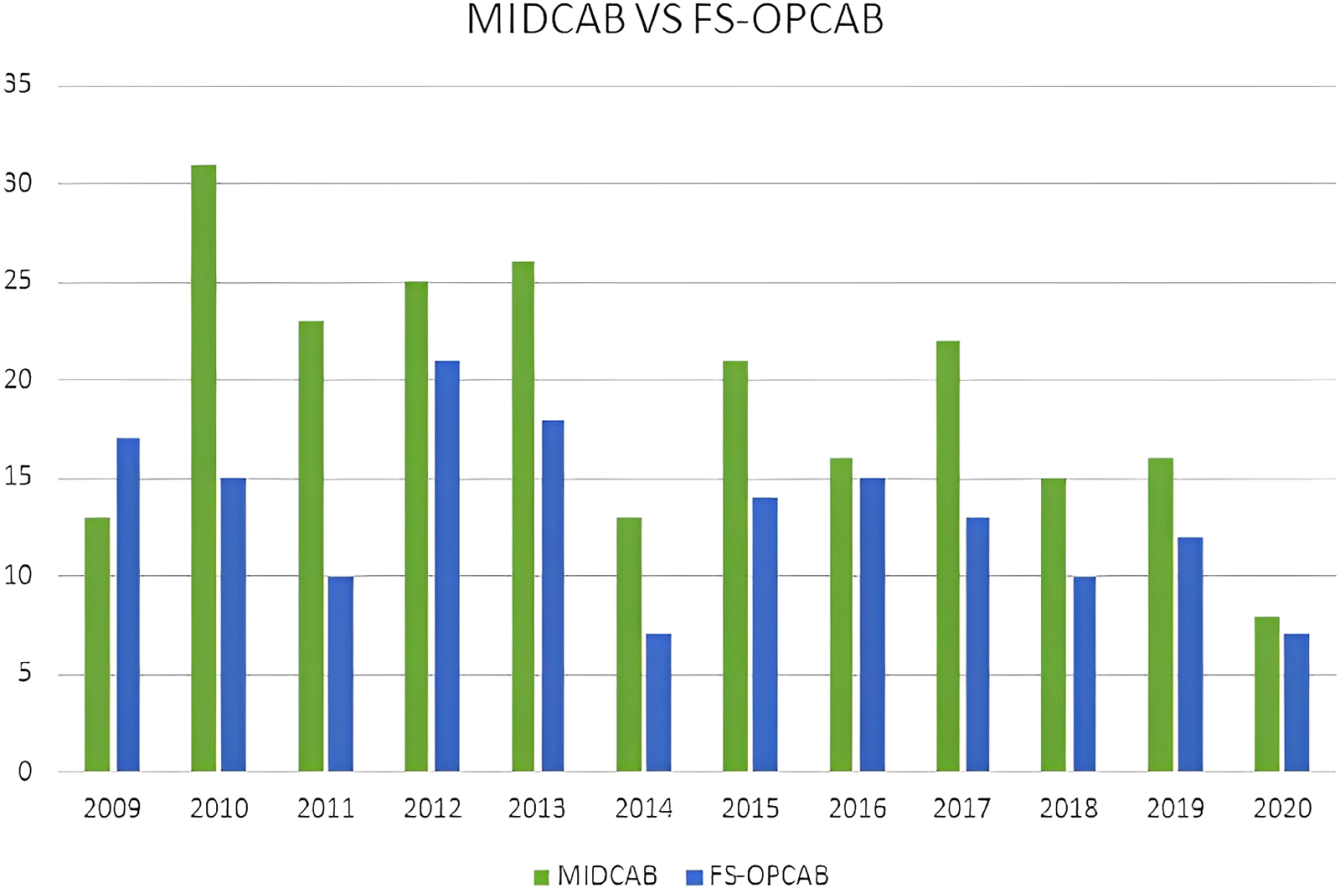
Number of MIDCAB and FS-OPCAB patients per year from 2009 to 2020.

A total of 388 patients were included in our retrospective study. Of these patients, 229 underwent MIDCAB through an antero-lateral minithoracotomy (MIDCAB group, mean age 62.7 ± 11.2 years), and 159 underwent off-pump myocardial revascularization via a median sternotomy (FS-OPCAB group, mean age 67.1 ± 10.3 years). Baseline characteristics are depicted in Table 1. The PS ranged from a low of 0.00000 to a high of 0.94949. The model was well calibrated among deciles of observed and expected risk (Hosmer–Lemeshow test *P* = 0.73). The discriminate power of the PS, as quantified by measurement of the receiver operating characteristics area, was found to be good before IPTW-adjustment (c-index 0.78, range 0.73–0.83) and was poor after IPTW-adjustment (c-index 0.46; range 0.42–0.50), indicating a well-balanced preoperative risk between the two IPTW-groups. The IPTW approach reduced the SMD in preoperative covariates between the study groups substantially. In the IPTW-groups, all standardized differences were <10%, with the exception of Euroscore II values. However, the difference in the two IPTW-groups was small, given the huge potential range of Euroscore II values. Over the study period the number of operations gradually decreased (Figure 2).

**Table 1:** Baseline characteristics in unweighted and weighted study population.

Parameter	FS *n* = 159	Unweighted patients *n* = 388 MIDCAB *n* = 229	SMD%	FS *n* = 377	Weighted *n* = 738 MIDCAB *n* = 361	SMD %
Age (years)^a^	67.1 ± 10.3	62.7 ± 11.2	40.9	64.1 ± 10.9	64.7 ± 11.6	−5.3
Sex, males^b^	103 (64.8)	169 (73.8)	−28.8	265 (70.3)	250 (69.3)	3
Body mass index (kg/m^2^)^b^	28.6 ± 4.5	27.5 ± 4.0	25.8	27.7 ± 4.4	27.9 ± 4.1	−4.7
Diabetes mellitus^b^	57 (35.8)	113 (24.4)	36.8	104 (27.6)	105 (29.4)	−5.5
Hypertension^b^	136 (85.5)	183 (79.9)	19.6	309 (82.0)	299 (82.8)	−3.0
Stroke^b^	3 (1.9)	6 (2.6)	−5.3	18 (4.8)	16 (4.4)	2.5
Myocardial infarction^b^	44 (26.7)	43 (23.1)	11.8	97 (25.7)	96 (26.5)	−2.5
COPD^b^	19 (11.9)	11 (4.8)	42.7	24 (6.4)	25 (6.9)	−2.6
Atrial fibrillation^b^	12 (7.5)	13 (5.7)	10.1	22 (5.8)	21 (5.8)	0
LVEF (%)^a^	56.0 ± 10.5	58.8 ± 8.5	−29.3	57.3 ± 9.2	58.0 ± 9.2	−7.6
eGFR (ml/min/1.73 m^2^)^a^	69.6 ± 21.8	79.6 ± 18.3	−49.7	75.6 ± 18.9	76.5 ± 20.4	−4.6
PAOD^b^	20 (7.5)	9 (3.9)	56.7	19 (5.0)	21 (5.8)	−4.5
Left main stenosis^b^	36 (22.6)	24 (10.5)	53.3	48 (12.7)	51 (14.1)	−5.5
Three vessel disease^b^	72 (45.3)	41 (17.9)	98.4	97 (25.7)	83 (22.9)	9.2
Euroscore II (%)^a^	2.79 ± 4.22	1.19 ± 1.04	52	1.41 ± 1.01	1.61 ± 1.85	−13.4
Aspirin use^b^	123 (77.4)	189 (82.5)	−18.9	308 (81.7)	292 (80.7)	3.6
Statin use^b^	94 (59.1)	152 (66.4)	−21.7	245 (65.0)	231 (64.0)	2.9
CHA_2_DS^b^ VASC Score^b^	2.87 ± 1.50	2.05 ± 1.37	57.1	2.36 ± 1.40	2.45 ± 1.62	−5.9
Current/previous Smokers^b^	74 (46.6)	100 (43.6)	8.5	170 (45.1)	172 (42.1)	8.5
Previous PCI^b^	58 (36.5)	89 (38.9)	−6.9	143 (37.9)	143 (39.6)	4.5
NYHA class^a^	2.28 ± 0.86	1.88 ± 0.77	49	1.99 ± 0.79	2.02 ± 0.78	−3.8

FS, full sternotomy; MIDCAB, minimally invasive direct coronary artery bypass; COPD, chronic obstructive pulmonary disease; LVEF, left ventricular ejection fraction; eGFR, estimated glomerular filtration rate; PAOD, peripheral arterial occlusive disease; PCI, percutaneous coronary intervention; NYHA, New York Heart Association; PS, propensity score; std. diff, standardized difference; CHA_2_DS_2_ VASC, score calculating stroke risk in patients with atrial fibrillation.

Values are mean ± SD or *n* (%). The table presents baseline patient characteristics among the primary study cohort and the propensity-matched cohort.

Propensity scores were calculated by logistic regression model with type of surgery (MIDCAB or full sternotomy) controlling for all patient covariates. The precision of discrimination and calibration of the PS were analysed with the c-statistic and the Hosmer–Lemeshow test. Inverse probability of treatment weighting (IPTW) to reduce the bias of unweighted estimators.

^a^ Mean and standard deviation.

^b^ Number and percentage.

### 6.2 Perioperative outcome

The perioperative complication rates in the MIDCAB group were lower or similar when compared to the FS-OPCAB group (Table 2). Selective double-lumen intubation was not possible in one of the MIDCAB candidates. The patient was intubated using a single lumen tube. As a result, minimal invasive access was no longer feasible and a conventional sternotomy was performed. Only one patient was converted into a full sternotomy due to inadequate LIMA flow and was revascularized using RIMA-to-LAD grafting. Only one patient in the MIDCAB group underwent graft revision in comparison to 3 graft revisions in the FS-OPCAB group. The operation time did not differ significantly between the MIDCAB und FS-OPCAB groups (133 min ± 28 vs. 136 min ± 38, *P* = 0.38). Less redo thoracotomies were necessary in the MIDCAB group (*n* = 13/3.6% vs. *n* = 30/8.0%, *p* = 0.012). Patients undergoing MIDCAB had fewer transfusions (0.93 units ± 1.83 vs. 1.61 units ± 2.52, *p* < 0.001), shorter mechanical ventilation time (7.6 ± 4.7 h vs. 12.1 ± 26.4 h, *p* = 0.005), and needed less hemofiltration (*n* = 0/0% vs. *n* = 8/2.4%, *p* = 0.004). No differences were observed in perioperative PCI rates (*n* = 9/2.5% vs. *n* = 5/1.3%, *p* = 0.29), occurrence of wound infections (*n* = 4/1.1% vs. *n* = 7/1.9%, *P* = 0.55), hospital stay (12.1 days ± 3.5 vs. 12.6 days ± 5.3, *p* = 0.12), or 30-day mortality (*n* = 0/0% vs. *n* = 3/0.8%, *p* = 0.25).

**Table 2:** Perioperative and postoperative outcomes in the IPTW-groups.

	FS *n* = 377	MIDCAB *n* = 361	*P*-value
Operation time (min)	136 ± 38	133 ± 28	0.38
Creatinine_max,_ mg/dl^a^	1.23 ± 1.07	1.06 ± 0.39	0.52
Troponin_max_^a^^,^^b^(pg/ml)	4,153 ± 16,862	838 ± 4,540	<0.001
hs troponin_max_^a^^,^^c^(ug/L)	1.24 ± 1.65	0.93 ± 1.82	<0.001
Red blood cell units	1.61 ± 2.52	0.93 ± 1.83	<0.001
Mechanical ventilation, hours^a^	12.1 ± 26.4	7.6 ± 4.7	0.005
Intensive care unit stay, days	1.82 ± 2.44	1.50 ± 2.79	0.1
Rethoracotomy (*n*, %)	30 (8.0)	13 (3.6)	0.012
Hemofiltration (*n*, %)	9 (2.4)	0 (0.0)	0.004
Stroke (*n*, %)	10 (2.6)	0 (0.0)	0.002
Myocardial infarction (*n*, %)	7 (1.9)	1 (0.3)	0.07
Perioperative stent implant (*n*, %)	5 (1.3)	9 (2.5)	0.29
Perioperative thoracic wound infection (*n*, %)	7 (1.9)	4 (1.1)	0.55
In-hospital stay, days^a^	12.6 ± 5.3	12.1 ± 3.5	0.12
Readmission for thoracic wound infection (*n*, %)	3 (0.8)	0 (0.0)	0.25
Thirty-day mortality (*n*, %)	3 (0.8)	0 (0.0)	0.25

FS, full sternotomy; MIDCAB, minimally invasive direct coronary artery bypass.

^a^ Mean with standard deviation.

^b^ *n* = 237 (FS group) and *n* = 187 (MIDCAB group).

^c^ *n* = 127 (FS group) and *n* = 178 (MIDCAB group).

**Figure 3:**
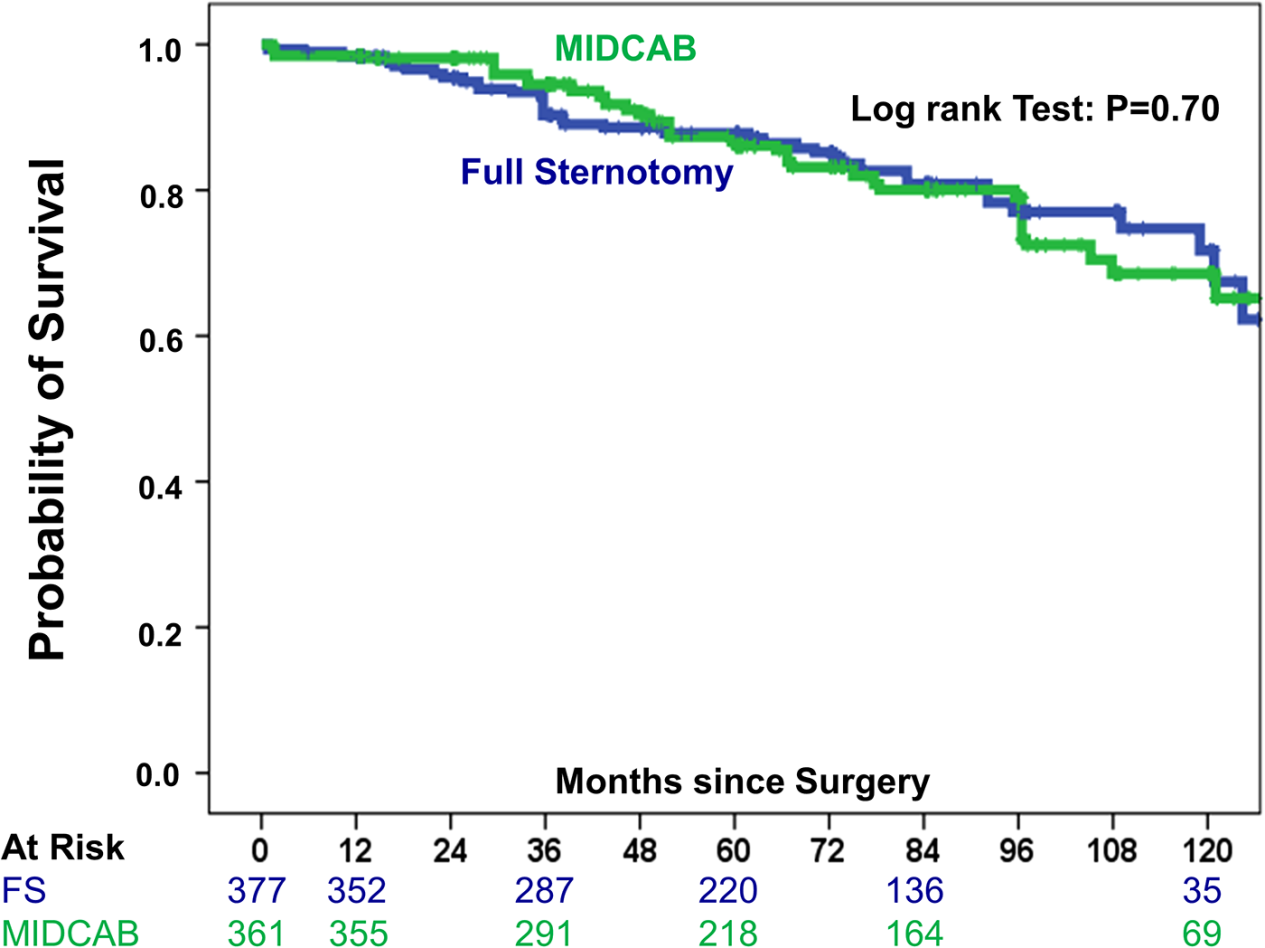
IPTW-adjusted Kaplan–Meier curve showing probability of survival of MIDCAB group (green line) vs FS-OPCAB group (blue line).

**Figure 4:**
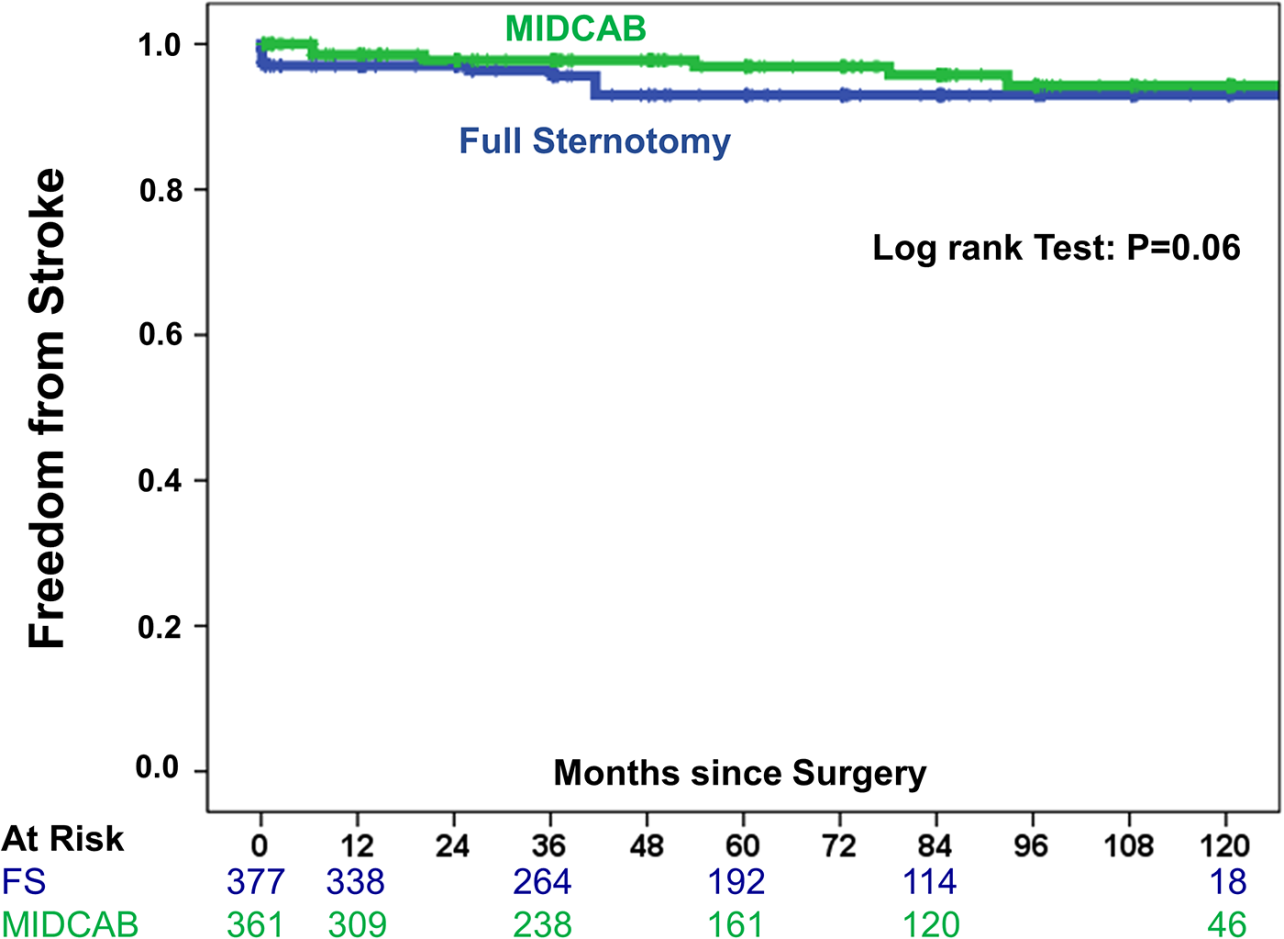
IPTW-adjusted Kaplan–Meier curve showing estimates of freedom from stroke of MIDCAB group (green line) vs. FS-OPCAB group (blue line).

**Figure 5:**
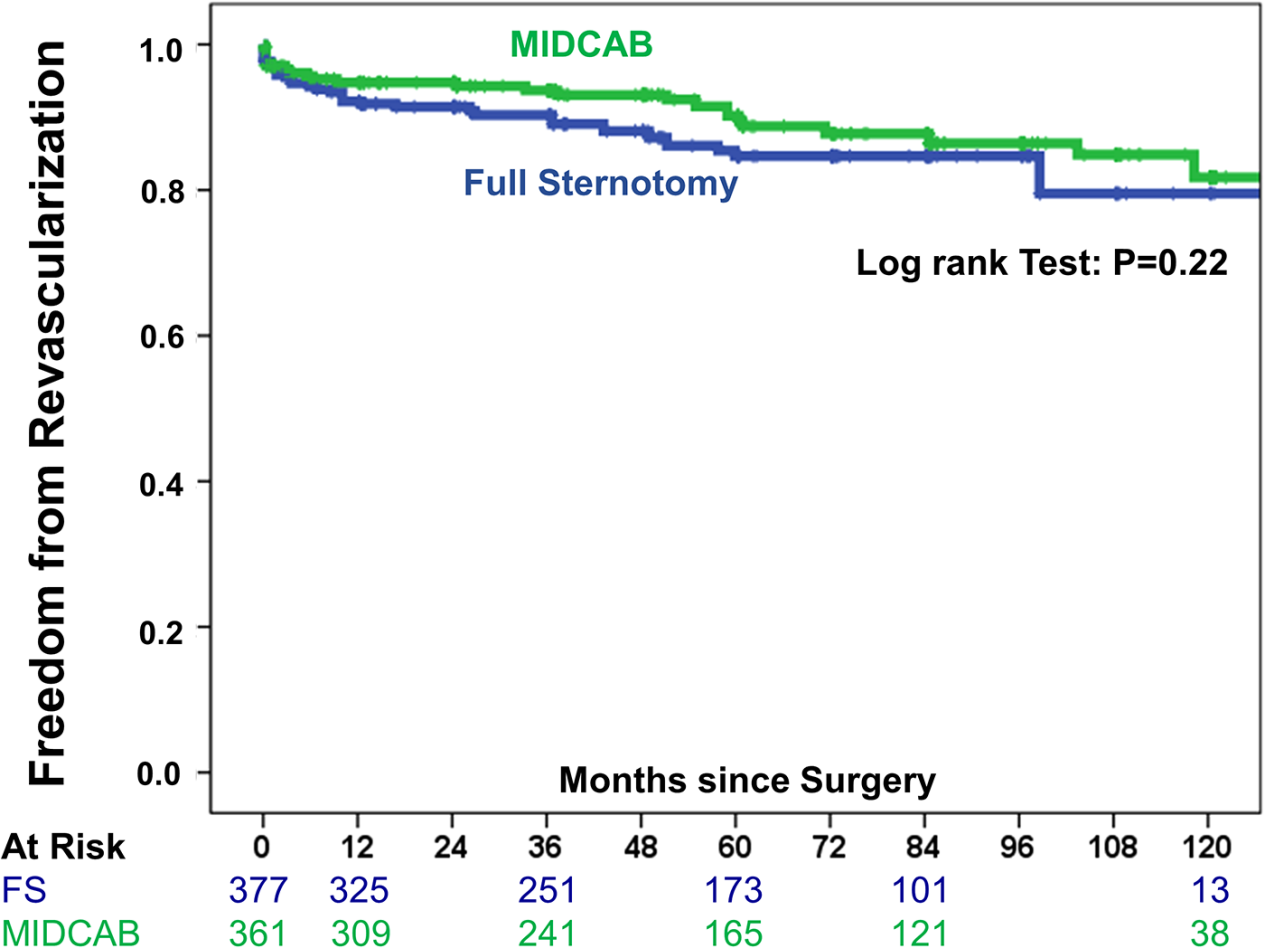
IPTW-adjusted Kaplan–Meier curve showing estimates of freedom from revascularization of MIDCAB group (green line) vs. full sternotomy group (blue line).

**Figure 6:**
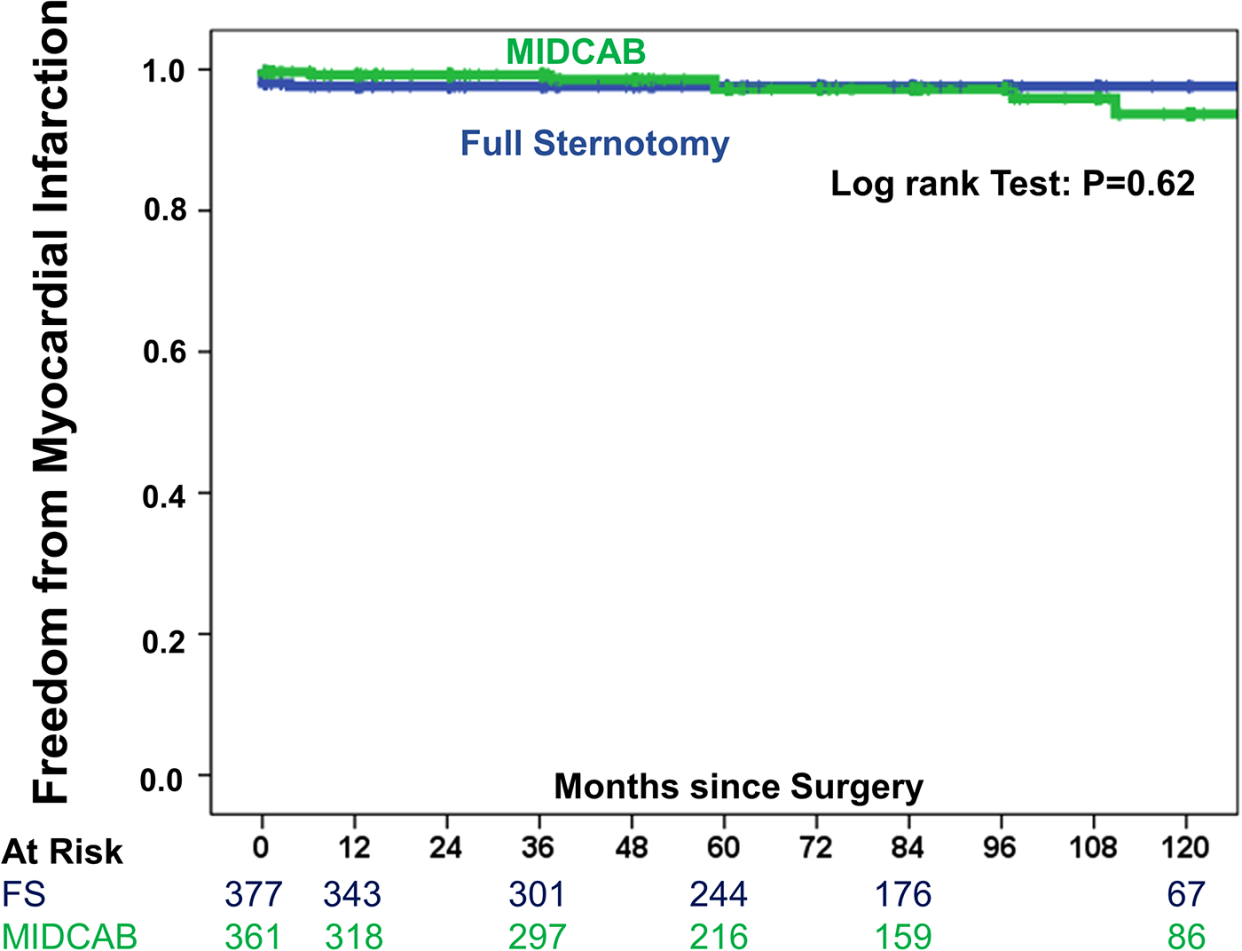
IPTW-adjusted Kaplan–Meier curve showing estimates of freedom from myocardial infarction of MIDCAB group (green line) vs. FS-OPCAB group (blue line).

To consider the issue of multiple testing, we applied the Benjamini and Hochberg FDR method to adjust the *P*-values. However, all respective results remained significant. Late mortality did not differ significantly between study groups (Figure 3). Briefly, in the FS-OPCAB group, the probability of survival at postoperative year 1, year 5 and year 10 was 98.4%, 87.8%, and 71.7%, respectively. In the MIDCAB group, the corresponding values were 98.4%, 87.7%, and 68.7%, respectively (RR 1.24, CI 0.87–1.86, *p* = 0.7). Results were consistent for 1-year and 5-year mortality between 2009–2014 and 2015–2020. In detail, the differences of 1-year and 5-year mortality between the MIDCAB and FS groups were for the 2009–2014 period 0.3% and 1.2%, respectively, and for the 2015–2020 period 0.2% and 1.0%, respectively. Likewise, the risk of stroke did not differ significantly between the two study groups (Figure 4). In the FS-OPCAB group, freedom from stroke at postoperative year 1, year 5 and year 10 was 97.0%, 93.0%, and 93.0%, respectively. In the MIDCAB group, the corresponding values were 98.5%, 96.9%, and 94.3%, respectively (RR 0.52, CI 0.25–1.09, *p* = 0.06). Freedom from repeat revascularization at postoperative year 1, year 5 and year 10 in the FS-OPCAB group was 92.2%, 84.7%, and 79.5%, respectively. In the MIDCAB group, the corresponding values were 94.8%, 90.2%, and 81.7%, respectively (RR 0.73, CI 0.47–1.16, *p* = 0.22). Results did not differ significantly between study groups (Figure 5). Freedom from myocardial infarct at postoperative year 1, year 5 and year 10 in the FS-OPCAB group was 97.6%, 97.6%, and 93.1%, respectively. In the MIDCAB group, the corresponding values were 99.2%, 97.6%, and 92.6%, respectively (RR 0.75, CI 0.31–1.81, *p* = 0.62). Freedom from myocardial infarct also did not differ significantly between study groups (Figure 6).

## 7 Discussion

MIDCAB surgery has been proposed as a less invasive alternative to full sternotomy revascularization in isolated LAD disease or in the context of hybrid revascularization strategies. Several reports have described low perioperative morbidity and mortality and excellent long-term survival (15–17). In the largest single-center cohort of a total of 2,667 patients who underwent MIDCAB surgery over a 22-year period—the longest follow-up reported so far—Davierwala and colleagues from the Leipzig group recently reported survival estimates of 88.0, 77.7, 66.1 and 55.6% at 5, 10, 15 and 20 years, respectively, which impressively was found to be better than the age- and sex-adjusted German population. Long-term results in the MIDCAB group of our study were comparable (15). Though, Davierwala et al. did not compare outcomes of MIDCAB patients with a full sternotomy group. Still, there are ongoing concerns that the minimally invasive approach may be associated with worse graft patency rates and a greater risk of repeat revascularization than OPCAB via sternotomy. In a best evidence systematic review conducted by Florisson et al. outcomes of patients who underwent MIDCAB or OPCAB for either single or multivessel disease were examined. From 187 identified studies, 12 were deemed most pertinent. MIDCAB patients had shorter ICU and total hospital length of stay length of stay. However, MIDCAB patients were more likely to experience early complications, urgent follow-up procedures, repeat revascularization events, progression of native disease, rehospitalization within three months, and postoperative myocardial infarction. They concluded, that MIDCAB is associated with greater morbidity and reintervention compared to OPCAB via sternotomy. Yet, there wasn't a noticeable rise in early or late mortality for MIDCAB in studies that had a mid-term follow-up (10). Similarly, Stanbridge and Hadjinikolaou conducted a meta-analysis comparing early studies of 3,304 MIDCAB cases with 3,060 OPCAB surgeries done via sternotomy. Both groups had comparable early death rates. While 5-year survival was similar [86.8% vs. 84.3% (*P* = 0.61)], pooled graft stenosis and occlusion rates showed a trend to be higher in the MIDCAB group (10.5% vs. 6.4%, *P *= 0.08). Of note, a significant reduction in the stenosis rates after the utilization of myocardial stabilizer devices was observed (12).

In the context of available MIDCAB literature, there is not only a paucity of data comparing the two different surgical approaches, especially when looking at risk-adjusted comparisons, but also with regard to longer-term follow-up outcomes. Moreover, advances in PCI technology, changes in revascularization guidelines and PCI practice, in addition to the adoption of MIDCAB have virtually abolished surgical revascularization of single-vessel disease via full sternotomy in patients with isolated LAD disease in daily cardiac surgical practice. Thus, making direct comparisons between these two surgical approaches is increasingly difficult. Therefore, in a risk-adjusted comparison, we juxtaposed the early postoperative and long-term results of MIDCAB vs. FS-OPCAB revascularization over a maximum follow-up period of 10 years. To our knowledge, our study is one of very few studies with rigorous baseline covariate adjustment to achieve a more reliable comparison between these two surgical approaches. In our study, MIDCAB was associated with lower perioperative complication rates when compared to FS-OPCAB revascularization. Patients in the MIDCAB group had less blood transfusion, lower postoperative Troponin peak values, and shorter time on ventilator, and lower need for surgical revisions than the FS-OPCAB group. These findings are consistent with other studies (18, 19). Notably, in our series there was no perioperative mortality in the MIDCAB group. Published results in the literature regarding early postoperative outcomes are somehow heterogenous and partly contradictory. In a study by Halkos et al., 147 patients who underwent MIDCAB/hybrid coronary revascularization were optimally matched in a 1:4 ratio with 588 OPCAB patients. MIDCAB patients exhibited a notably higher rate of subsequent revascularization events (12.2% vs. 3.7%, *P* < 0.001), target vessel treatments (8.8% vs. 3.1%, *P* = 0.002), progression in the original disease (4.8% vs. 0.9%, *P* < 0.001), and issues with the internal mammary artery (4.8% vs. 1%, *P* < 0.001) (11). Similarly, Vicol found that MIDCAB surgery was related to a significantly higher incidence of occluded or stenosed anastomoses and necessity for immediate reintervention (20). This is contradictory to our observations.

In a study including 668 patients, with 508 undergoing MIDCAB and 160 receiving full sternotomy CABG, Raja et al. showed comparable 30-day mortality (2.0% vs. 2.5%), stroke rates (1.3% vs. 1.4%), and repeat revascularization rates (0.8% vs. 1.3%). Preoperative demographics and risk profile of both groups were comparable. Long-term survival rates, tracked over a mean follow-up time of 12.95 ± 0.45 years were comparable between the two groups. The authors observed 153 deaths with 113 (22.24%) in MIDCAB group and 40 (25%) in the full sternotomy group with *p* = 0.64 (21).

In another recent retrospective analysis spanning two decades, Mastroiacovo et al. evaluated long-term outcomes of 141 patients who underwent MIDCAB and 133 patients who underwent LIMA-to-LAD grafting via a full median sternotomy. Mean follow-up was 133 and 98 months, respectively. However, extent of disease was different in both groups. In their series 35.5% and 52.5% of MIDCAB and FS-OPCAB patients had multivessel-disease, respectively. The survival trajectory over this period was promising, starting at 100% at the 1-year mark and tapering to 70% after 20 years. Similarly, the freedom from MACCE encompassing myocardial infarction, stroke, and cardiac death, began at 97% in the first year and declined to 61% by the end of the study period. The MIDCAB group experienced fewer in-hospital stays, required fewer blood transfusions, and had a reduced incidence of cardiac-related events than their counterparts in the full sternotomy group. However, the reported results were not adjusted to baseline covariates (18).

Detter and colleagues compared outcomes from 129 MIDCAB and 127 OPCAB patients. Early postoperative angiography (7.4 ± 5.8 days) revealed consistent rates of graft functionality or significant stenoses >50%. Of note, patients in the OPCAB group were significantly older, with higher Canadian cardiovascular society (CCS) classification, a lower LVEF and more challenging anatomies (22).

The findings of the mentioned studies underscore the equipoise or even potential advantages of MIDCAB, not only in terms of survival and MACCE but also in the context of post-operative recovery and overall prognosis when compared to full sternotomy bypass procedures. In this context, Davierwala et al. observed that despite an increasing number of urgent and emergent cases and the worsening risk profiles of patients, the in-hospital mortality remained constantly low throughout the study period and the cumulative log likelihood ratio of observed-to-expected mortality rate dropped to less than 1 after 1,000 procedures and decreased further to less than 0.5 after 1,600 procedures. Concordantly, graft revisions dropped over the study period from 4.4% to 0.8% (15). Obviously, these findings suggest that MIDCAB is technically more demanding and emphasizes that it should be reserved for specialized and highly dedicated, experienced surgeons. In the report by Xu et al., postoperative evaluations using coronary CTA or vessel bridge angiography revealed occlusions in both the OPCAB and MIDCAB groups (13.3% vs. 7.8%). The findings indicated a vessel graft patency rate over 92% in the MIDCAB group, compared to 87% in the OPCAB group (19).

With regard to long-term patency rate, a recent trial conducted by Guo et al. studying 566 patients who underwent minimally invasive coronary artery bypass grafting, including multiple grafting, at 12 years, the cumulative incidence of repeat revascularization was 14.8% ± 2.5%, and the cumulative incidence of repeat revascularization for graft failure was 5.4% ± 2.8% (23). Furthermore, the Swedish Web System for Enhancement and Development of Evidence-Based Care in Heart Disease Evaluated According to Recommended Therapies registry published in a study encompassing 1,939 patients who underwent isolated LAD revascularization CABG, with a mean follow-up period of 17.2 years, that only a little more than one third of these patients (38.6%) underwent clinically-driven postoperative angiography within 20 years after the surgery. Among these angiographies, ITA graft failure was identified in only 16.4% of cases which equates to roughly 6% of the total patient population (24). In our study, we reported a low rate of clinical adverse events which is consistent with these findings.

MIDCAB surgery claims to reduce the surgical trauma and to improve cosmesis. Indeed, the necessity for blood transfusions was significantly lower in the MIDCAB group when compared to the full-sternotomy group. Nevertheless, Ng and colleagues reported a significant higher rate of wound complications after MIDCAB surgery as compared to the sternotomy approach (25). Similarly, Detter et al. and Raja et al. observed a higher incidence of postoperative wound infections in the MIDCAB group (21, 22), a finding that we could not observe. However, the potential for wound infections after MIDCAB should be taken into consideration.

## 8 Study limitations

The most important limitation of the present study is its retrospective and nonrandomized design which certainly limits the generalizability of our results. Even PS-based baseline covariate adjustment cannot exclude unmeasured or unknown confounders and despite extensive adjustment, selection bias cannot be ruled out completely. In addition, we did not adjust for the underlying anatomical complexity of coronary lesions (i.e., presence of long-segment calcifications, tortuosity, chronic total occlusion) within the two different groups, which may have influenced surgical access choice and hence results. Additionally, we were unable to identify the primary cause of death and could not discriminate between cardiac and non-cardiac mortality. Beyond the equipoise between MIDCAB and FS-OPCAB in the assessed endpoints, we can therefore only speculate about the quality of revascularization. Most importantly, we were not able to analyze long-term graft patency and the incidence of graft failure since follow-up coronary angiography was not performed systematically. Additionally, no follow-up data was available to compare the groups with respect to freedom from angina and freedom from repeat target vessel revascularization events. Lastly, factors such as surgeon's experience and learning curve effects were not considered. To verify the results of this study and to draw more robust conclusions, a randomized multi-central clinical trial with a larger patient number and the systematic performance of postoperative coronary angiographies is warranted.

## 9 Conclusions

In conclusion, our findings demonstrate the safety and efficacy of MIDCAB, without an increased risk of early or late mortality, repeat revascularization, or myocardial infarction, even during extended follow-up periods of up to 10 years, compared to off-pump full sternotomy LIMA-to-LAD grafting. Importantly, our study also highlights reduced risks of perioperative complications for patients undergoing MIDCAB. Given these outcomes, cardiac surgeons should consider incorporating MIDCAB more confidently into their practice, particularly in hybrid revascularization settings. Our study, therefore, not only contributes to the current body of knowledge but also aims to dispel some of the prevailing uncertainties surrounding MIDCAB, advocating for its wider acceptance and utilization in the treatment of coronary artery disease.

## Data Availability

The raw data supporting the conclusions of this article will be made available by the authors, without undue reservation.
