# Supplementary figures and images for: Early and late outcomes after minimally invasive direct coronary artery bypass vs. full sternotomy off-pump coronary artery bypass grafting
Source: Front Cardiovasc Med. 2024 Feb 21;11:1298466. doi: 10.3389/fcvm.2024.1298466 (PMC10914960; doi:10.3389/fcvm.2024.1298466)

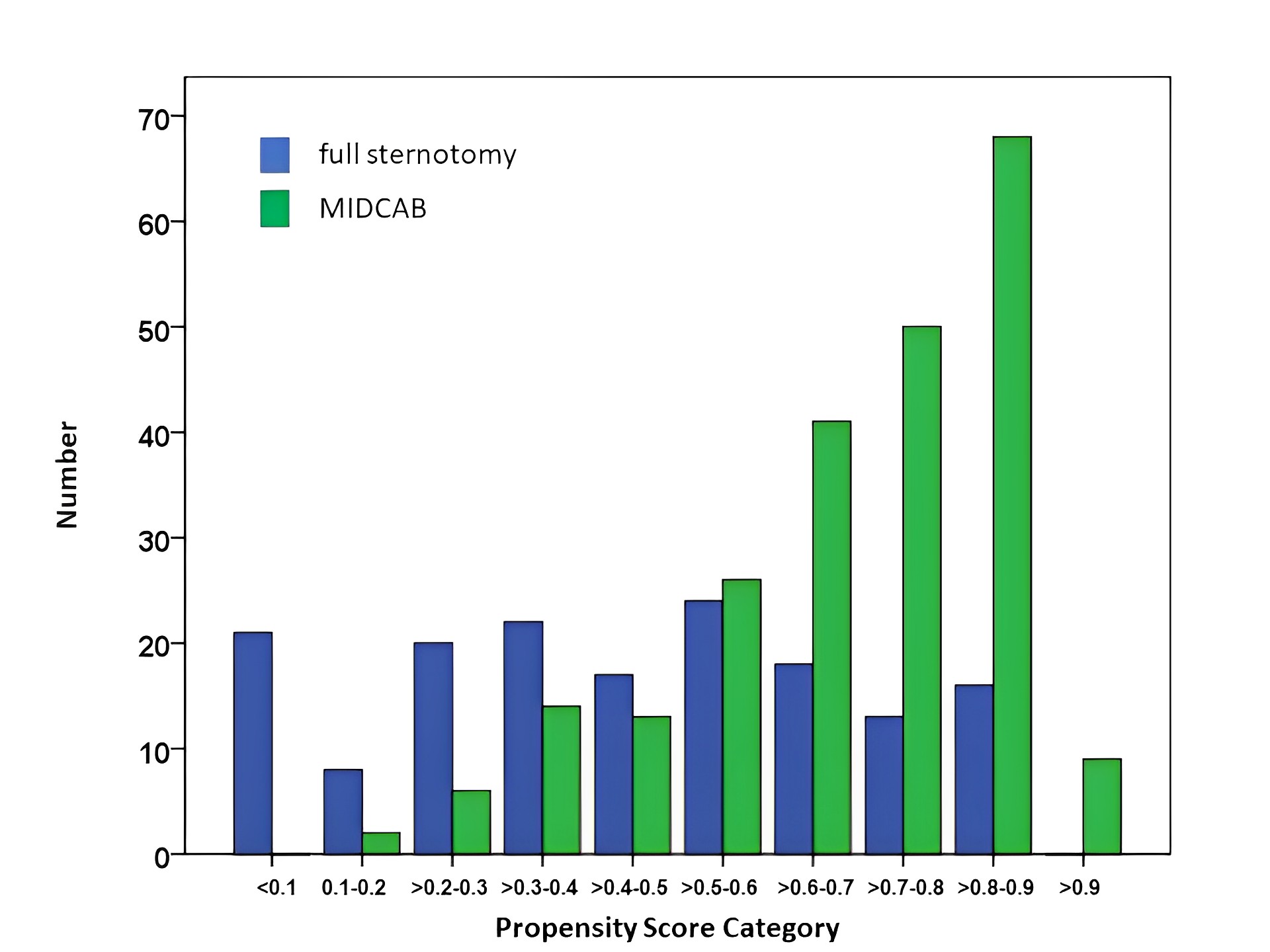

Supplement: Supplementary file 1 [file Image1.jpeg]
